# An Optimized Flutter-Driven Triboelectric Nanogenerator with a Low Cut-In Wind Speed

**DOI:** 10.3390/mi12040366

**Published:** 2021-03-29

**Authors:** Yang Xia, Yun Tian, Lanbin Zhang, Zhihao Ma, Huliang Dai, Bo Meng, Zhengchun Peng

**Affiliations:** 1 Key Laboratory of Optoelectronic Devices and Systems of Ministry of Education and Guangdong Province, College of Physics and Optoelectronic Engineering, Shenzhen University, Shenzhen 518060, China; xyxiaxy@gmail.com (Y.X.); 1910454039@email.szu.edu.cn (Y.T.); mzh_930930@163.com (Z.M.); zcpeng@szu.edu.cn (Z.P.); 2 School of Energy and Power Engineering, University of Shanghai for Science and Technology, Shanghai 200093, China; 3 Department of Mechanics, Huazhong University of Science and Technology, Wuhan 430074, China; zhanglb1993@foxmail.com (L.Z.); daihulianglx@hust.edu.cn (H.D.)

**Keywords:** triboelectric nanogenerator, flutter-driven, energy harvesting, wind energy

## Abstract

We present an optimized flutter-driven triboelectric nanogenerator (TENG) for wind energy harvesting. The vibration and power generation characteristics of this TENG are investigated in detail, and a low cut-in wind speed of 3.4 m/s is achieved. It is found that the air speed, the thickness and length of the membrane, and the distance between the electrode plates mainly determine the PTFE membrane’s vibration behavior and the performance of TENG. With the optimized value of the thickness and length of the membrane and the distance of the electrode plates, the peak open-circuit voltage and output power of TENG reach 297 V and 0.46 mW at a wind speed of 10 m/s. The energy generated by TENG can directly light up dozens of LEDs and keep a digital watch running continuously by charging a capacitor of 100 μF at a wind speed of 8 m/s.

## 1. Introduction

With the environmental problems and energy crisis becoming more serious, it is necessary to find various green and renewable energy sources [1]. Among the various green energy sources in nature, wind energy has attracted much attention, due to the advantages of its abundance and wide distribution [2]. In addition, with the rapid development of portable and miniaturized electronic devices, the demand for self-powered and continuous power sources is increasing. Energy harvesting technologies based on piezoelectric [3,4], electrostatic [5,6], and electromagnetic [7,8] mechanisms have been well explored and widely applied. 

In the past few years, triboelectric nanogenerator, which operates based on triboelectrification and electrostatic induction effects, has been greatly developed and has shown great potential as a promising approach for effective energy harvesting and active sensing [9,10]. The triboelectric nanogenerator (TENG) has the advantages of high performance, simple manufacture, light weight, and low cost. As reported in a large number of studies, TENGs have been successfully employed to harvest mechanical energy from ambient and daily life, such as wind, sound, fluid, and human motions [11,12,13,14,15,16,17,18,19,20,21,22,23,24,25,26,27,28,29,30,31,32,33,34,35,36,37,38].

Regarding wind energy harvesting, there are mainly two categories of TENGs, which are rotational structures [23,24,25,26,27,28] and flutter-driven structures [29,30,31,32,33,34,35,36,37]. Among them, flutter-driven TENG (FTENG) usually adopts a simple structure and is easy to be designed with electronic systems. Yang et al. [23] firstly reported a flutter-driven TENG composed of two parallel Al layers and a one-end-fixed FEP film to harvest wind energy. An open-circuit voltage of 100 V and a power density of 29.09 mW/m^2^ were obtained under a wind speed of 10 m/s. Ravichandran et al. [36] developed a flutter-driven TENG with a maximum output power density of 2850 mW/m^2^, which improves the output performance of FTENG.

However, the onset wind speed of the existing FTENGs is still at a relatively high value, usually over 5 m/s. In this work, we developed an optimized FTENG with a low onset wind speed of 3.4 m/s, and the output voltage of this FTENG reaches about 100 V at the onset wind speed. The vibration and power generation characteristics of the TENG are studied in detail.

## 2. Results and Discussion

### 2.1. Design and Working Mechanism of FTENG

**Figure 1:**
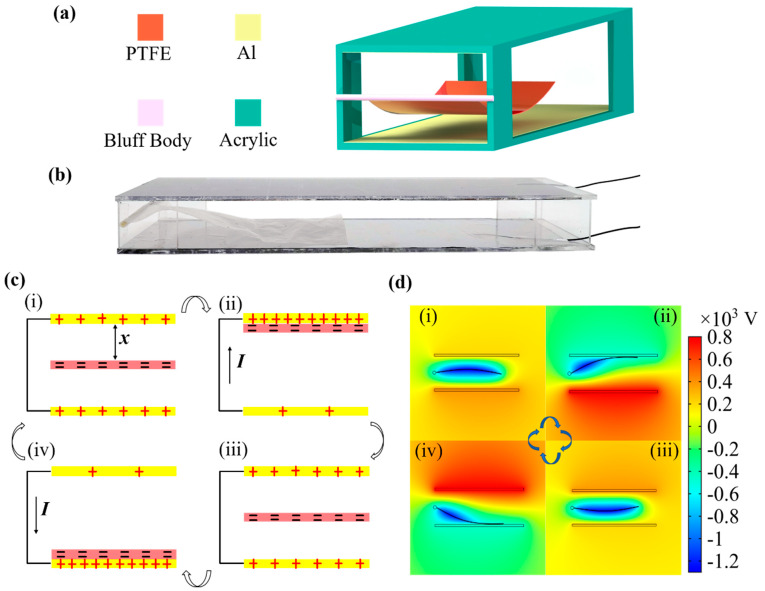
(**a**) Schematic of the flutter-driven triboelectric nanogenerator (FTENG), (**b**) Photograph of the FTENG, (**c**) Schematic of the working mechanism of FTENG, and (**d**) Finite-element simulation of the electrostatic potential distribution of the FTENG.

Figure 1a illustrates a schematic diagram of the FTENG. It is composed of two parallel Al electrodes and a one-end-fixed PTFE film. Two 30 μm thick Al films are attached to acrylic plates and fixed by four small rectangular acrylic pillars to form a wind channel. The PTFE film is then stuck on a bluff body with a diameter of 0.8 mm and attached to one end of the wind channel. The width of the channel is 5 cm. A photograph of a fabricated FTENG is shown in Figure 1b.

When the air flows through the rigid channel at a certain speed, the PTFE film will oscillate and periodically contact and separate with the Al electrodes. When the flexible film alternately oscillates between the two parallel electrodes due to wind-induced fluttering, the TENG generates alternating electric current under triboelectrification and electrostatic induction effects. The working principle of a single cycle is shown in Figure 1c. Due to the difference in electron-attracting abilities between the two materials, electrons will be transferred from the surface of Al to PTFE, when they are brought into contact with each other. After several cycles of contact and separation, the triboelectric charge on the surfaces of the PTFE film and the Al electrodes will be saturated. When the PTFE film vibrates upwards, the positive charge of the bottom electrode will flow along the external circuit to the top electrode to balance the potential difference between the two electrodes. When the film continues to move downward, the positive charge of the top electrode will flow back to the bottom electrode. Finally, the film will return to the initial position and again reaches electrostatic equilibrium. To further understand the potential change in FTENG, a finite element method simulation of the potential distribution under open-circuit conditions was performed using COMSOL Multiphysics (V5.4, COMSOL), as shown in Figure 1d. Since the area of Al is larger than the area of PTFE, the surface charge density of PTFE was set to be 10 μC/m^2^, and the surface charge density of Al electrode was set to be 8 μC/m^2^. When the PTFE film moves between the two Al electrodes, the open-circuit voltage (VOC) changes accordingly. Therefore, when the two electrodes are externally connected to the circuit, the alternating current is generated as a result of VOC changing, which drives charge back and forth between the two electrodes.

### 2.2. Vibration Characteristics of the FTENG

**Figure 2:**
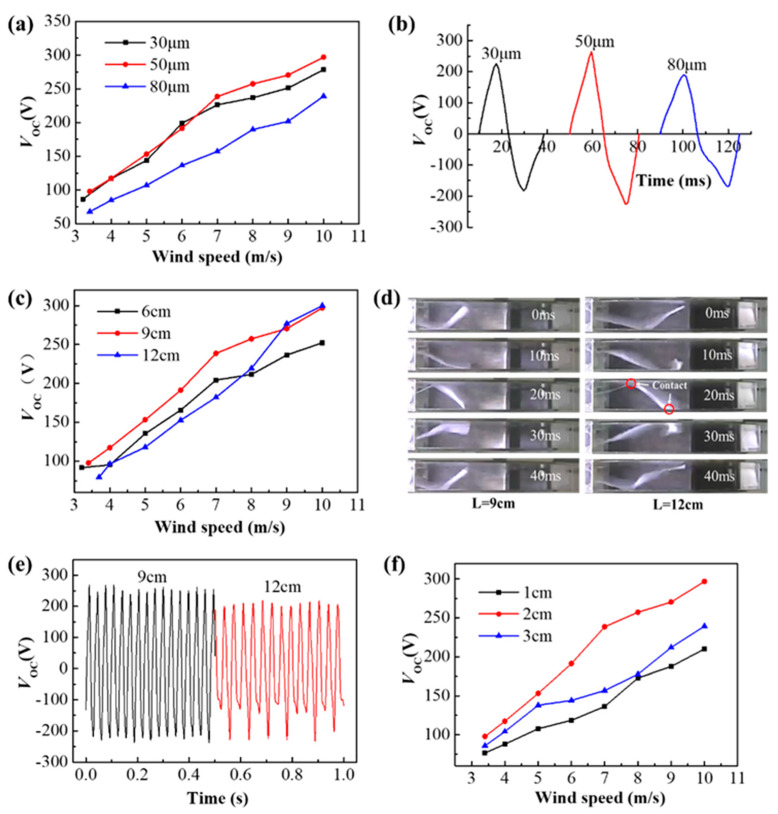
(**a**) Open-circuit voltage corresponding to three thicknesses of *h* = 30 μm, 50 μm, and 80 μm at different wind speeds. (**b**) Single-cycle *V*_OC_ waveform of three thickness films when the wind speed is 8 m/s. (**c**) Open-circuit voltage corresponding to three lengths of *L* = 6 cm, 9 cm, and 12 cm at different wind speeds. (**d**) Vibration photographs of a single-cycle for film lengths of *L* = 9 cm and *L* = 12 cm at a wind speed of 8 m/s. (**e**) the waveform diagram of *V*_OC_ at *L* = 9 cm and *L* = 12 cm at a wind speed of 8 m/s for 0.5 s. (**f**) Open-circuit voltage corresponding to electrode plate distances of *d* = 1 cm, 2 cm, and 3 cm at different wind speeds.

In order to optimize the power output of the FTENG, it is necessary to deeply understand the flutter behavior of the PTFE film. Figure 2a shows the section view of the film vibrating, in which the fluid velocity *U*, the length *L,* and the thickness *h* of the flexible film are marked. It is assumed that the deformation of PTFE film is two-dimensional and that the viscoelastic damping of the material and the tension due to the viscous boundary layers are neglected. As discussed in previous studies [35,39,40], the motion of the film is mainly determined by the wind speed *U*, the film length *L*, and the flexural rigidity *D* (related to Young’s modulus *E*, Poisson’s ratio *θ*, and thickness *h* of the material), the fluid density *ρ_a_* and the film density *ρ_f_*. Since the fluid is air at normal temperature and pressure, and the flexible film’s material is PTFE, *ρ_a_*, *ρ_f_*, *E* and *θ* are constants. Therefore, the variables considered for the PTFE film motion are mainly the wind speed *U*, film length *L*, and film thickness *h*.

Although the PTFE film is confined to a rigid channel in this work, which is more complicated than the previously discussed unconstrained cases, the film fluctuation in the unconstrained situation can still be approximated and used in the rigid channel [33]. Therefore, the main factors affecting the fluctuation of the flexible film are the wind speed *U*, the film length *L*, the film thickness *h*, and, additionally, the distance *d* between the two electrode plates.

### 2.3. Multi-Parameter Optimization of FTENG

As discussed above, to improve the output performance, the structural optimization of FTENG mainly considers the three parameters of film length *L*, thickness *h*, and the distance *d* between the two electrodes.

#### 2.3.1. Effect of the Thickness *h*

When the distances between the electrode plates and the film length are chosen as 2 cm and 9 cm, the open-circuit voltage *V*_OC_ corresponding to different wind speeds is measured at three different thicknesses of 30 μm, 50 μm, and 80 μm, as shown in Figure 2a. It can be seen that PTFE film with a thickness of 80 μm has the lowest open-circuit voltage at the same wind speed. When the wind speed is less than 7 m/s, the voltages of 30 μm and 50 μm are approximately the same. With the wind speed exceeds 7 m/s, the voltage of 50 μm is larger than that of 30 μm. Vibration frequencies of the three films are 35 Hz, 32 Hz, and 28 Hz, respectively, when the wind speed is 8 m/s. Figure 2b shows the single-cycle *V*_OC_ waveform. The periods *T* corresponding to the thicknesses of 30 μm, 50 μm, and 80 μm are 28.6 ms, 30.7 ms, and 35 ms, respectively. It can be seen that as the thickness increases, the cycle time also increases. Since the distance *d* between the electrode plates is 2 cm, the average moving speeds (*v* = 2*d*/*T*) of the three thickness films between the electrode plates are 1.4 m/s, 1.3 m/s, and 1.14 m/s, respectively. Assuming that the average speed of film motion is *v*_1_ and the instantaneous speed *v*_2_ after contact with the electrode is 0 m/s, the collision force between the film and the electrode can be obtained by the momentum theorem (*Ft* = *mv*_2_ − *mv*_1_). The collision force of the mass per unit surface of the film is simplified as *F* = −*ρ_f_ hv*_1_/*t*, where *t* is the instantaneous time when the film collides with the electrode and “−” indicates that the motion of the film is opposite to the direction of the force. Assuming that the time *t* at the instant of film collision with the electrode is the same, the collision forces corresponding to the film thickness of 30 μm, 50 μm, and 80 μm are 4.2e^−5^*ρ_f_*/*t*, 6.5e^−5^*ρ_f_*/*t*, 9.12e^−5^*ρ_f_*/*t*, respectively, and the unit is N. Therefore, the thicker the PTFE film, the greater the contact force generated between the film tail and the Al electrode.

The power generation performance has a great relationship with the contact force and effective contact area. However, the thicker the film, the larger the flexural rigidity *D*, so it is difficult to form an effective fit when the film is in contact with the electrode, and the effective contact area is very small. Therefore, in comparison with three different thickness films, the 80 μm thickness film corresponds to the lowest voltage at different wind speeds. Before the wind speed of 7 m/s, the combined effects of the contact force and contact area of the 50 μm and 30 μm thickness films are almost the same, so the difference between the open-circuit voltages of the two thickness films is small. After the wind speed exceeds 7 m/s, the tail of 50 μm thickness has a greater contact force with the electrode, and can effectively fit when it is in contact with the electrode, so the voltage is higher than that of 30 μm in thickness. Therefore, a film thickness of 50 μm is more appropriate.

#### 2.3.2. Effect of Length *L*

When the distance between the electrode plates and the film thickness are chosen as 2 cm and 50 μm, the open-circuit voltage *V*_OC_ corresponding to different wind speeds *U* is measured at three different lengths of 6 cm, 9 cm, and 12 cm, as shown in Figure 2c. It can be seen that when *L* = 9 cm, the corresponding overall voltage is the highest in the wind speed measurement range and only slightly decreases at the last high wind speed. It is considered that since the contact area of the film is small when *L* = 6 cm, the voltage is not as high as that at *L* = 9 cm. When *L* = 12 cm, the film is easy to contact with upper and lower electrodes at the same time during the vibration process, which is not conducive to the charge transfer between the two aluminum electrodes, so the open-circuit voltage *V*_OC_ is lower. This weakening effect is very obvious at low wind speeds, and it can be observed that the *V*_OC_ when *L* = 12 cm is the smallest of the three lengths when the wind speed is less than 8 m/s. The single-period vibration photographs of PTFE films of *L* = 9 cm and *L* = 12 cm were taken at a wind speed of 8 m/s with a high-speed camera (GC-PX100, JVC, Japan) at a frame rate of 500 Hz, as shown in Figure 2d. It is evident that it is easier to simultaneously contact the upper and lower electrodes when *L* = 12 cm, which is disadvantageous for charge transfer and leads to a reduction in the potential difference between the two electrodes. Figure 2e shows the waveform diagram of *V*_OC_ at *L* = 9 cm and *L* = 12 cm, and it can be clearly seen that the voltage is larger at *L* = 9 cm. It can also be seen that the troughs of *V*_OC_ have a large fluctuation at *L* = 12 cm. This is due to the long length of the PTFE film, which causes the tail end to roll up during the vibration easily and be unable to effectively contact the electrode.

#### 2.3.3. Effect of Distance *d* between Electrode Plates

Finally, when the PTFE film length and the film thickness were chosen as 9 cm and 50 μm, the open-circuit voltage VOC corresponding to different wind speeds U was measured at three distances between the electrode plates of *d* =1 cm, 2 cm, and 3 cm, as shown in Figure 2f. It is evident that *d* = 2 cm corresponds to the maximum open-circuit voltage at different wind speeds. The larger the electrode distance, the smaller the contact area with the electrode plate is when the film vibrates. Therefore, the *V*_OC_ corresponding to *d* = 2 cm is larger than that of *d* = 3 cm. However, since the electrode of *d* = 1 cm is too narrow, the film is more likely to be in contact with the two electrodes at the same time during the vibration process, and the potential between the electrode plates is smaller. Therefore, the plate distance *d* should not be too large or too small, and it is more suitable to take 2 cm.

According to the above analysis, when the film thickness *h*, length *L,* and distance *d* of electrode plates are 50 μm, 9 cm, and 2 cm, respectively, a higher open-circuit voltage can be obtained.

**Figure 3:**
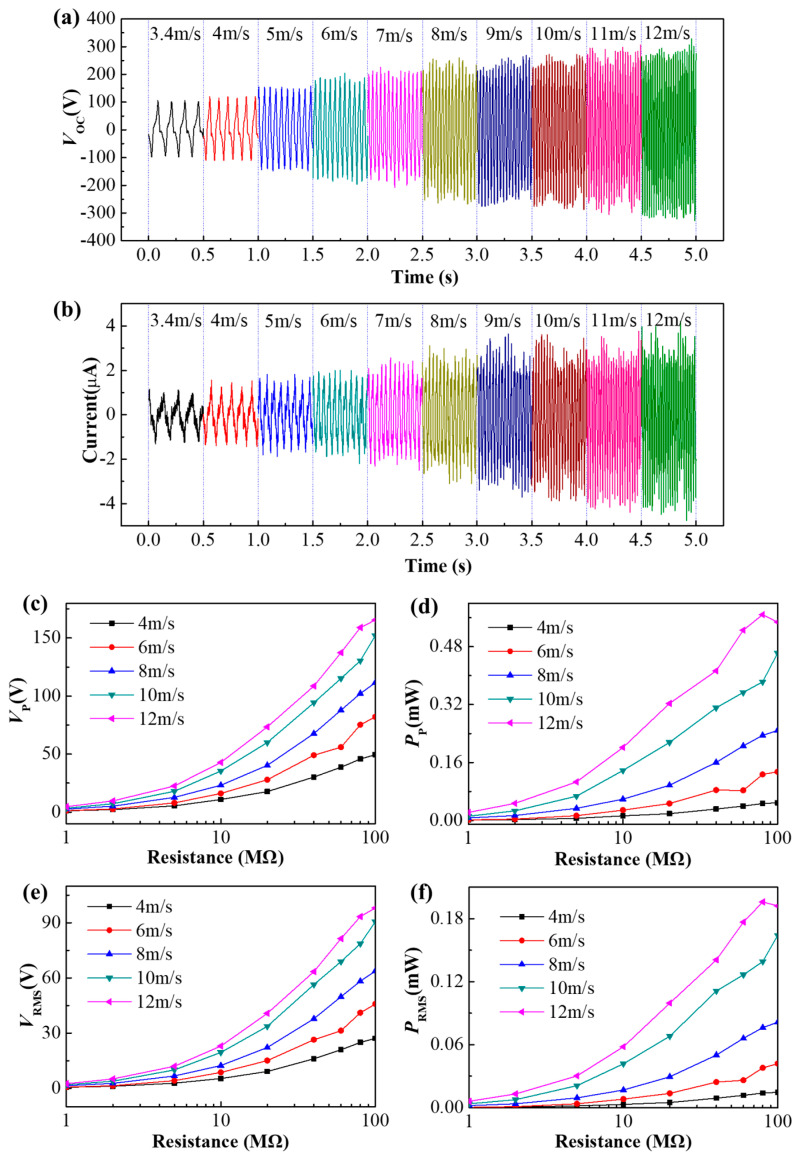
(**a**,**b**) Time histories of open-circuit voltage and current for different wind speeds. (**c**–**f**) Electrical outputs of FTENG varying with load resistance: (**c**) Peak voltage *V*_P_, (**d**) Peak power *P*_P_, (**e**) Average voltage *V*_RMS_, (**f**) Average power *P*_RMS_.

To make the surface of the film accumulate a sufficient amount of charge, the TENG is initially stimulated at a wind speed of 10 m/s for 5 min. The time history curves of the open-circuit voltage *V*_OC_ and the current measured using a sampling resistor of 100 kΩ at different wind speeds are shown in Figure 3. It can be seen that as the wind speed increases, the open-circuit voltage and current increase significantly. In addition, the frequency of the waveforms gradually increases from 8 Hz at the wind speed of 3.4 m/s to 48 Hz at the wind speed of 12 m/s. The higher wind speed leads to a much high vibration frequency of the membrane. Thus, a higher output voltage and power could be achieved. When the wind speed is 10 m/s, the maximum value of *V*_OC_ reaches 297 V, and the current is 3.9 μA. The peak voltage *V*_P_ and peak power *P*_P_ of the FTENG depend on the external load ranging from 1 MΩ to 100 MΩ at different wind speeds, which are shown in Figure 3c,d. The average voltage *V*_RMS_ and the average power *P*_RMS_ are shown in Figure 3e,f. It can be seen that as the wind speed *U* increases, the voltage, current, and power increase. When the wind speed *U* is 10 m/s, the maximums of *P*_P_, *V*_P_, *P*_RMS_, *V*_RMS_ are 0.46 mW, 152 V, 0.16 mW, and 91 V with a load of 100 MΩ, respectively.

### 2.4. Performance of the Optimized FTENG

**Figure 4:**
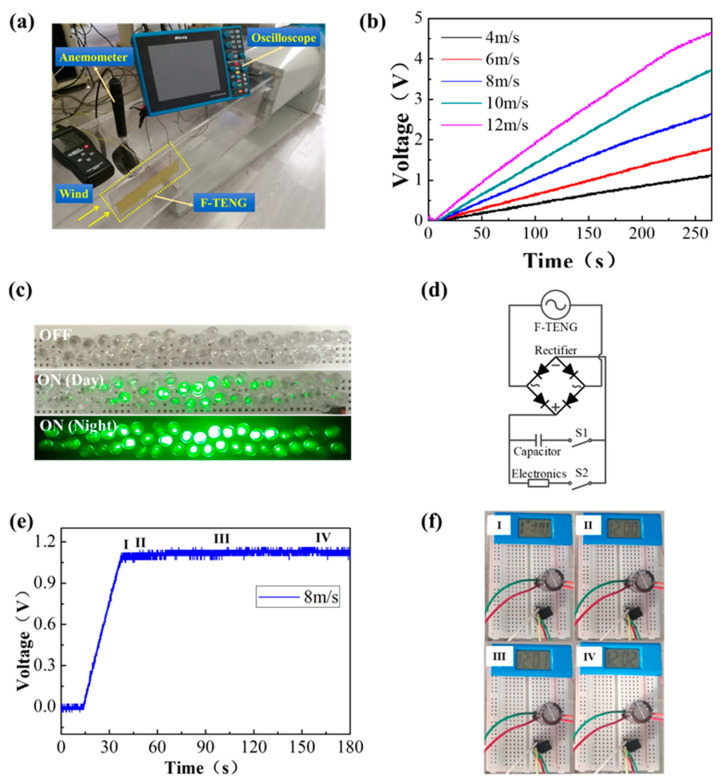
(**a**) The measurement and application setup of FTENG, (**b**) Charging a 100 μF capacitor at different wind speeds, (**c**) Lighting up dozens of LEDs by FTENG, (**d**) The equivalent circuit for powering electronic devices via a capacitor, (**e**,**f**) Continuously powering a digital watch.

The electrical energy generated by FTENG can be rectified to charge energy storage devices for powering various electronic systems. Figure 4a shows the measurement and application setup of FTENG. Figure 4b shows the charging process for a 100 μF capacitor at different wind speeds. The higher the wind speed, the faster the charging speed. The FTENG directly lights up dozens of LEDs at a wind speed of 8 m/s, as shown in Figure 4c. Figure 4d shows the equivalent circuit for powering electronic devices by charging a capacitor. The FTENG can continuously power a digital watch, as shown in Figure 4e,f. After closing the two switches simultaneously, the FTENG charges a 33 μF capacitor. When the capacitor voltage reaches a specific value, the digital watch will be driven to work. It is seen that after 38 s of charging, the digital watch starts to work, and the voltage s is maintained at about 1.09 V. After a few seconds, the digital watch displays the time normally and keeps working continuously.

## 3. Conclusions

In summary, an optimized flutter-driven TENG has been developed and characterized in detail. By changing the thickness and size of the film and the distance between electrode plates to achieve multi-parameter optimization, this work systematically analyzes and obtains an optimized design with lower cut-in wind speed and massive power output. When the film thickness, length, and distance between the electrode plates being 50 μm, 9 cm, and 2 cm, respectively, a cut-in wind speed of 3.4 m/s is obtained. The open-circuit voltage of FTENG reaches 297 V at a wind speed of 10 m/s. The output power reaches 0.46 mW, and the power density is 127.78 mW/m^2^. Finally, the practical applications of the optimized FTENG are demonstrated by lighting up a few dozen commercial LEDs and keeping a digital watch working continuously, showing an outstanding application potential.

## 4. Experimental Methods

Characterization of the device: a handheld oscilloscope (TO1102, Micsig, China) was employed to measure the output voltage with a 100:1 oscilloscope probe, and the short-circuit current was measured using a sampling resistor of 100 kΩ. The uniform airflow is generated and controlled by a wind tunnel, and an anemometer (AS8336, SMART SENSOR, China) is employed to measure the wind speed.

## Data Availability

The data presented in this study are available on request from the corresponding author.
